# COVID‐19: Histopathological correlates of imaging patterns on chest computed tomography

**DOI:** 10.1111/resp.14101

**Published:** 2021-06-22

**Authors:** Azar Kianzad, Lilian J. Meijboom, Esther J. Nossent, Eva Roos, Bernadette Schurink, Peter I. Bonta, Inge A. H. van den Berk, Rieneke Britstra, Jaap Stoker, Anton Vonk Noordegraaf, Paul van der Valk, Erik Thunnissen, Marianna Bugiani, Harm Jan Bogaard, Teodora Radonic

**Affiliations:** ^1^ Department of Pulmonary Medicine, Amsterdam Cardiovascular Sciences Amsterdam UMC, Vrije Universiteit Amsterdam Amsterdam The Netherlands; ^2^ Department of Radiology and Nuclear Medicine, Amsterdam Cardiovascular Sciences Amsterdam UMC, Vrije Universiteit Amsterdam Amsterdam The Netherlands; ^3^ Department of Pathology, Cancer Centre Amsterdam Amsterdam UMC, Vrije Universiteit Amsterdam Amsterdam The Netherlands; ^4^ Department of Pulmonary Medicine Amsterdam UMC, AMC Amsterdam The Netherlands; ^5^ Department of Radiology and Nuclear Medicine Amsterdam UMC, AMC Amsterdam The Netherlands

**Keywords:** acute respiratory distress syndrome, chest CT, coronavirus disease, COVID‐19, histopathological and imaging, SARS‐CoV‐2, viral infection

## Abstract

**Background and objective:**

Patients with coronavirus disease 2019 (COVID‐19) pneumonia present with typical findings on chest computed tomography (CT), but the underlying histopathological patterns are unknown. Through direct regional correlation of imaging findings to histopathological patterns, this study aimed to explain typical COVID‐19 CT patterns at tissue level.

**Methods:**

Eight autopsy cases were prospectively selected of patients with PCR‐proven COVID‐19 pneumonia with varying clinical manifestations and causes of death. All had been subjected to chest CT imaging 24–72 h prior to death. Twenty‐seven lung areas with typical COVID‐19 patterns and two radiologically unaffected pulmonary areas were correlated to histopathological findings in the same lung regions.

**Results:**

Two dominant radiological patterns were observed: ground‐glass opacity (GGO) (*n* = 11) and consolidation (*n* = 16). In seven of 11 sampled areas of GGO, diffuse alveolar damage (DAD) was observed. In four areas of GGO, the histological pattern was vascular damage and thrombosis, with (*n* = 2) or without DAD (*n* = 2). DAD was also observed in five of 16 samples derived from areas of radiological consolidation. Seven areas of consolidation were based on a combination of DAD, vascular damage and thrombosis. In four areas of consolidation, bronchopneumonia was found. Unexpectedly, in samples from radiologically unaffected lung parenchyma, evidence was found of vascular damage and thrombosis.

**Conclusion:**

In COVID‐19, radiological findings of GGO and consolidation are mostly explained by DAD or a combination of DAD and vascular damage plus thrombosis. However, the different typical CT patterns in COVID‐19 are not related to specific histopathological patterns. Microvascular damage and thrombosis are even encountered in the radiologically normal lung.

## Graphical abstract

We present direct regional comparison of the most frequently observed pulmonary computed tomography (CT) patterns with corresponding histopathological patterns in an autopsy cohort of eight coronavirus disease 2019 (COVID‐19) patients. While different stages of diffuse alveolar damage are key tissue substrates of the CT patterns in COVID‐19, vascular damage and thrombosis contribute to these typical radiological findings as well.

**See related** Editorial

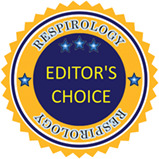

## INTRODUCTION

The ongoing pandemic of coronavirus disease 2019 (COVID‐19) poses a global challenge, with over 135 million confirmed cases and over 2.9 million deaths at the time of writing (World Health Organization weekly report 11 April). Clinical presentation ranges from mild symptoms such as fever, dry cough and shortness of breath to a fatal disease with development of acute respiratory distress syndrome (ARDS).^1^, ^2^ Chest computed tomography (CT) is widely used in the diagnosis and management of COVID‐19.^3^ Typical chest CT findings are found in approximately 85% of patients with severe or non‐severe disease^2^ and sometimes even in patients before the onset of symptoms.^4^ An integrated understanding of the histopathology that underlies typical imaging features in COVID‐19 is currently lacking. Demonstration of a strict correlation between imaging and tissue patterns could form the basis for imaging‐guided patient management, for example, directing choices for anti‐inflammatory or anti‐fibrotic treatment in COVID‐19.

Typical COVID‐19 CT patterns include bilateral peripheral ground‐glass opacities (GGOs) with or without consolidation, crazy paving, band‐like consolidations, thickened interlobular septa and traction bronchiectasis.^5^ In early disease, GGO is more common than consolidation but there is a significant overlap of radiological patterns at different disease stages.^4^ It is assumed that different stages of diffuse alveolar damage (DAD) are key tissue substrates of the different CT patterns in COVID‐19.^6^, ^7^, ^8^, ^9^ In several COVID‐19 autopsy cohorts, endothelialitis associated with the presence of the intracellular virus and thrombotic microangiopathy^10^ were described in addition to DAD.^11^, ^12^ This COVID‐19 vasculotropism resulting in endothelialitis, thrombosis and angiogenesis is a unique feature of COVID‐19, setting the disease apart from other viral pneumonias. However, it is unknown if and how this vascular damage pattern contributes to the typical radiological findings in COVID‐19. In this study, we present the first direct regional correlation of imaging findings to corresponding histological patterns in an autopsy cohort of eight COVID‐19‐infected patients. Our study aims to improve the interpretation of CT patterns, with the ultimate goal to provide image‐based guidance to diagnostic and therapeutic strategies.

## METHODS

### Patient selection

We performed a prospective autopsy study at the Amsterdam University Medical Centre. Patients were included during the first wave of COVID‐19 in the Netherlands, between 1 March and 10 May 2020. Inclusion criteria were (1) positive COVID‐19 PCR, (2) chest CT performed in the 72 h prior to death and (3) absence of clinical evidence of comorbidities that may result in lung disease other than COVID‐19. All patients fulfilling these criteria were included in the study.

### Chest CT

Chest CT was performed on three multidetector CT scanners: Siemens Somatom Force (Siemens Healthineers, Erlangen, Germany), Siemens Somatom Drive and a GE Discovery 750 HD (GE Healthcare, Milwaukee, MI). All patients underwent CT scanning of the chest in the supine position during end‐inspiration. Seven patients had chest CT without intravenous contrast medium and one patient with intravenous contrast medium (iobitridol, Xenetix® 300, Guerbet Laboratories, Roissy, France). Slice thickness for all scanners was between 0.625 and 1.25 mm. HD lung (GE Healthcare) kernel, pulmonary Br59F kernel (Siemens Somatom Drive) or pulmonary BI57d (Siemens Somatom Force) were applied. In four patients, at the same time, a CT pulmonary angiogram was performed for the clinical suspicion of pulmonary embolism.

To quantify pulmonary involvement on chest CT, Syngo.via CT Pneumonia software analysis program (Siemens Healthineers) was used. Chest CT findings were described according to international standard nomenclature defined by the Fleischner Society glossary.^13^ All CT images were reviewed by two expert pulmonary radiologists at the same time in the same sessions. Decisions were reached by consensus.

### Autopsies and gross examination of the lungs

**FIGURE 1:**
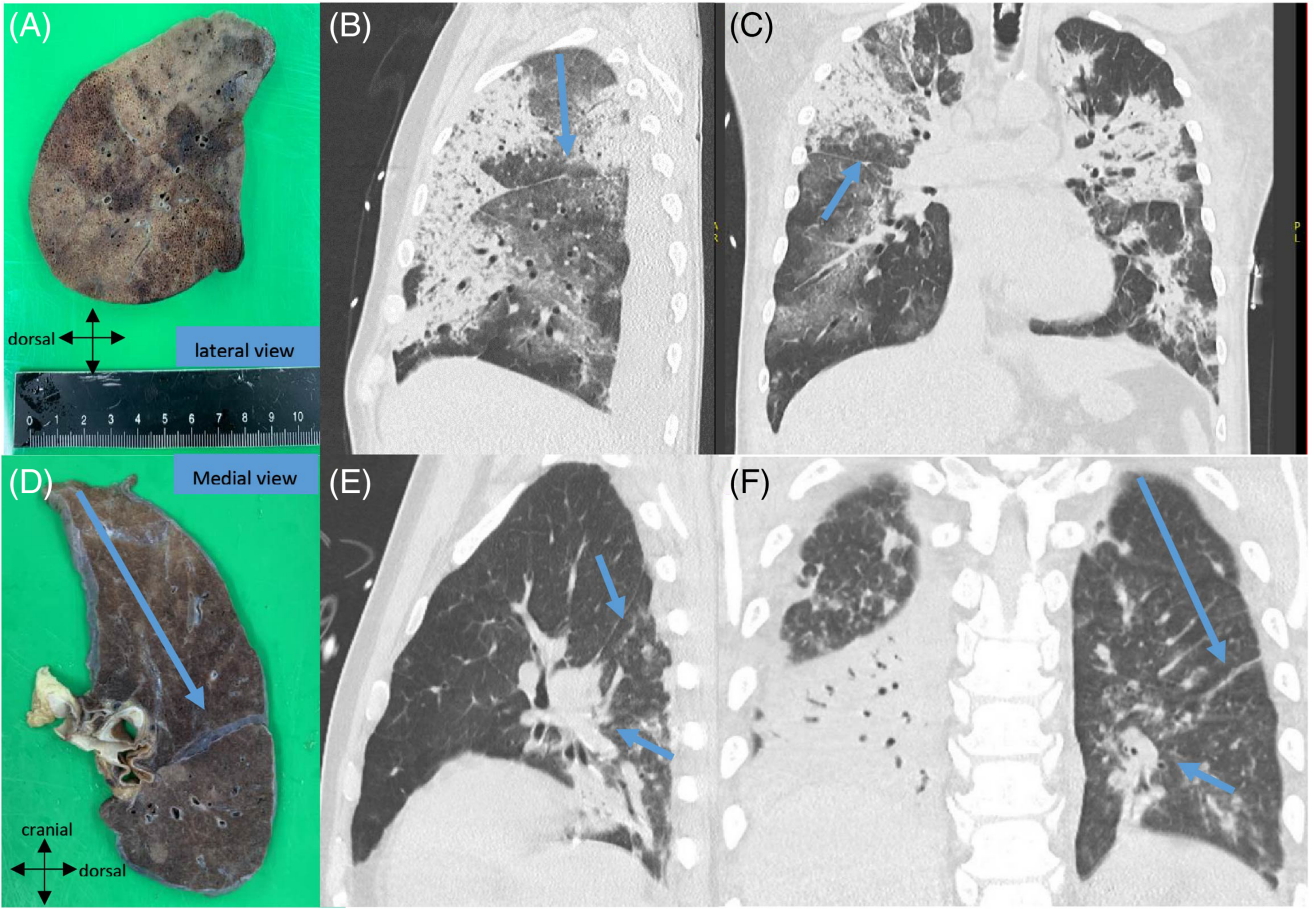
Fresh resection specimen of right lung photographed from the lateral view (A). Distance from the lobar apex and fissure relevant to the CT‐pattern of interest is measured on the CT scan and subsequently in the specimen in order to reach exactly the same level (B,C). (D) Another example of the left lung photographed from the medial view which correlates with the premortem CT image (E,F) in order to correlate with the oblique fissure and bronchial branch (arrows)

All autopsies were performed at the Department of Pathology of the Amsterdam University Medical Centre. Autopsy was performed within 24 h of death in order to ensure the optimal preservation of tissues. For fixation, the whole right or left lung was infused with 10% neutral buffered formalin via the main bronchus and subsequently submerged in that fixative for at least 48 h. Then, 5‐mm thick slices were cut in the transversal craniocaudal plane. Gross examination and sampling for microscopical examination of the lung was performed by a specialized lung pathologist as described before^14^ in order to ensure the direct correlation with the chest CT. In short, the pathologist begins with orientation of the specimen, maintaining the three‐dimensional (3D) anatomical orientation (in axial, sagittal and coronal planes). Distance from the lobar apex, fissure or bronchial branch relevant to the CT pattern of interest is measured on the CT scan and subsequently in the specimen in order to reach exactly the same level. Specimen is photographed from the medial and lateral direction delineating the 3D orientation (Figure 1) on the images.

### Integrative case evaluation

All cases were evaluated in joint sessions in the presence of a lung pathologist, chest radiologist and a pulmonologist. For every patient, correlation was performed of lung slices during gross examination with the chest CT. After extensive CT‐guided sampling, a total of 27 areas with typical COVID‐19 CT findings and two radiologically unaffected pulmonary areas were correlated to regional histopathological findings.

### Histopathology

Tissue was embedded in paraffin and 3 μm slides were cut and stained with haematoxylin and eosin. For Elastica‐van‐Gieson staining, slides were deparaffinized and rehydrated up to 70% alcohol, incubated for 60 min in Lawson solution, differentiated in 100% and 96% alcohol, rinsed, stained with Mayer's haematoxylin for 5 min, rinsed for 5 min, incubated with van Gieson picrofuchsin for 5 min and dehydrated in graded ethanol and xylene.

### Statistical analysis

Data that were normally distributed are presented as means (SD); data outside the normal distribution are presented as medians (minimum and maximum ranges). Categorical variables were summarized as counts and percentages. All data were analysed with R, version 3.6.3 (R Foundation for Statistical Computing, Vienna, Austria).

## RESULTS

### Patient population

Eight patients were included in this study. Patient characteristics are described in Table 1. Three patients appeared to be infected during hospitalization for other medical reasons. One patient was admitted due to an acute haematological malignancy and he suffered from severe pancytopenia and neutropenia. Median time (range) between CT and death was 1 (0–3) day. In cases with multiple CT examinations, the last premortem scan was selected for analysis. The onset from COVID‐19 symptoms until death ranged from 5 to 44 days, with the shortest disease course duration for the three already hospitalized patients probably due to pre‐existing conditions. All previously described typical COVID‐19 radiological patterns were found: patchy and diffuse GGO (thickened interlobular and/or intralobular septa) and different types of consolidation (Table 2). More atypical radiological patterns (e.g., reversed halo sign and nodules) were not observed.

**TABLE 1:** Baseline patient characteristics

Patients (*n*)	8
Male sex, *n* (%)	7 (88)
Age, years	65.6 ± 11.2
Comorbidity, *n* (%)	
Cardiovascular disease	3 (38)
Asthma	1 (13)
Neurological disease	1 (13)
Malignancy	2 (25)
Long‐term medication, *n* (%)	
Anti‐hypertensive drugs	2 (25)
Statins	3 (28)
Diabetes drugs	1 (13)
Antiplatelet drugs	2 (25)
Symptoms, *n*	
Fever	6
Cough	5
Dyspnoea	2
Headache	2
Other	2
Duration of COVID‐19 symptoms until death, days (range)	15.5 (5–44)
Pulmonary embolism, *n* (%)	3 (38)
Signs of RV dysfunction, *n*	
Histology	0
CTPA	
RV/LV > 1	2
Enlarged pulmonary artery	4
Cause of death, *n* (%)	
COVID‐19 respiratory failure	4 (50)
Multi‐organ failure	2 (25)
Malignancy	1 (12.5)
Neurological disease, not related to COVID‐19	1 (12.5)

Abbreviations: COVID‐19, coronavirus disease 2019; CTPA, computed tomography pulmonary angiogram; LV, left ventricle; RV, right ventricle.

**TABLE 2:** Correlation of radiological patterns with tissue damage patterns

Radiological patterns	Clinical histopathological features	Duration of symptoms (days)
1. Normal/spared pulmonary parenchyma	Focal vascular damage and thrombosis: endothelialitis/microthrombi/microhaemorrhages	5–22
2A. GGO patchy	Patchy vascular damage and thrombosis: endothelialitis/microthrombi/microhaemorrhages	6–22
	Exudative stage DAD	22
	Proliferative (organization of exudate) stage DAD	22
2B. GGO diffuse with thickened interlobular septa	Vascular damage and thrombosis: segmental infarction/microthrombi/microhaemorrhages	14
	Exudative stage DAD	14–44
	Proliferative (organization of exudate) stage DAD	44
2C. GGO with thickened interlobular and/or intralobular septa (crazy paving)	Exudative stage DAD	10
3A. Consolidation (subpleural/peribronchovascular) with or without surrounding GGO	Vascular damage and thrombosis: large haemorrhage/microthrombi/microhaemorrhages	5–22
	Exudative stage DAD	10–22
	Proliferative (organization of exudate) stage DAD	17
	Bronchopneumonia	5–6
3B. Consolidation sharply demarcated	AFOP	14
3C. Consolidation (band‐like) with subpleural sparing or bronchiectasis	Proliferative (organization of exudate) stage DAD	14–44
	Fibrotic stage DAD	30–44

Abbreviations: AFOP, acute fibrinous and organizing pneumonia; DAD, diffuse alveolar damage; GGO, ground‐glass opacity.

### Comparing histopathology with radiological patterns

Detailed correlation of the observed radiological and histopathological patterns is given in Table 2, Tables S1 and S2, Appendix S1 and Figures S1–S8 in the Supporting Information. We highlight the distinguishing COVID‐19 correlations in detail in the following paragraphs.

### Tissue substrates of GGO: DAD, vascular damage and thrombosis

**FIGURE 2:**
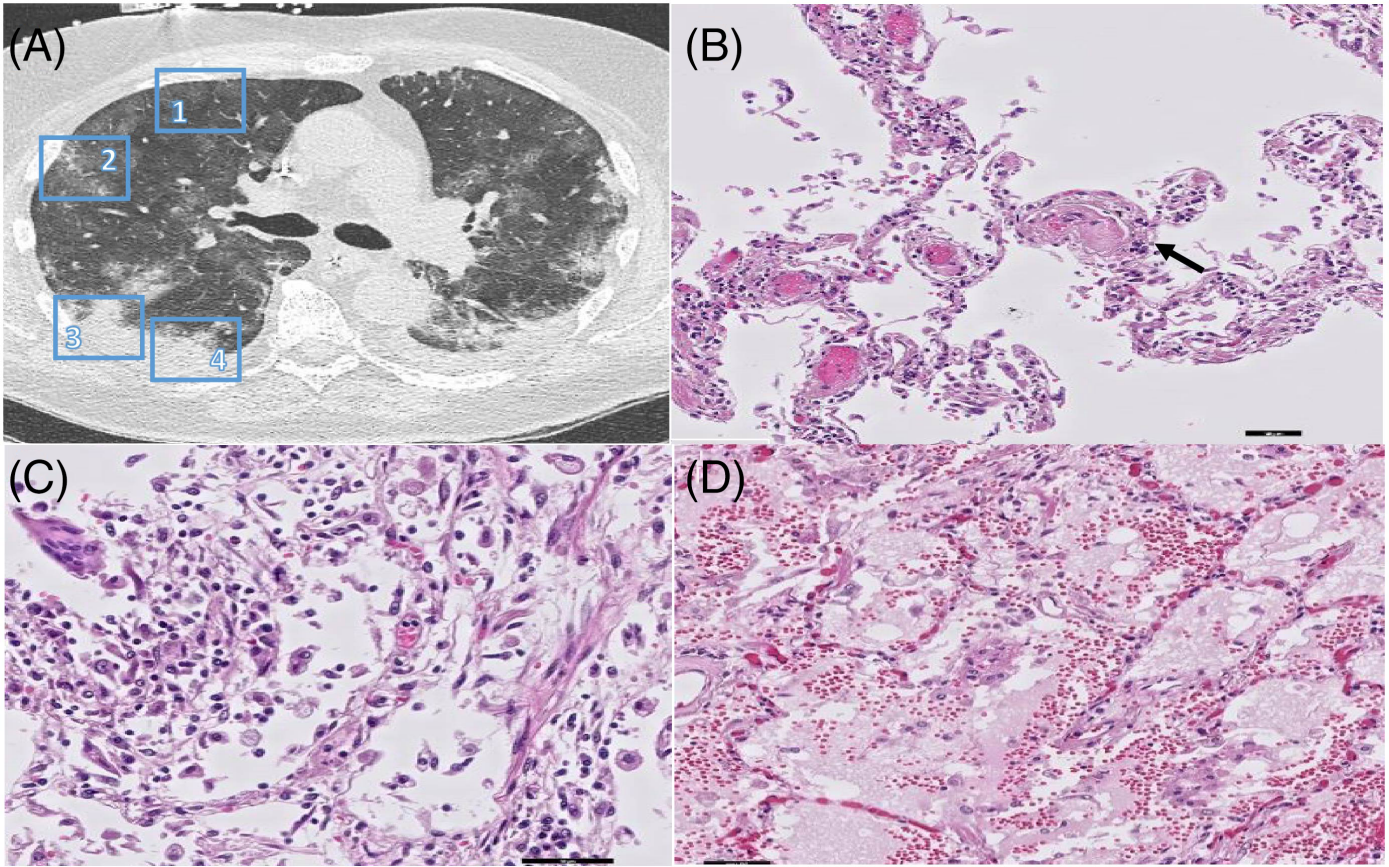
(A) CT image of the right lung reveals areas of Patchy GGO (region 1 & 2) and Consolidation with surrounding GGO (region 3 and 4). (B) Corresponding histopathology of Patchy GGO (region 1 and 2) revealed prominent microthrombi (arrow) and (C) patchy stage DAD. (D) Corresponding histopathology of Consolidation with surrounding GGO (region 3 and 4) revealed exudative DAD (scale bars correspond to 50 μm)

**FIGURE 3:**
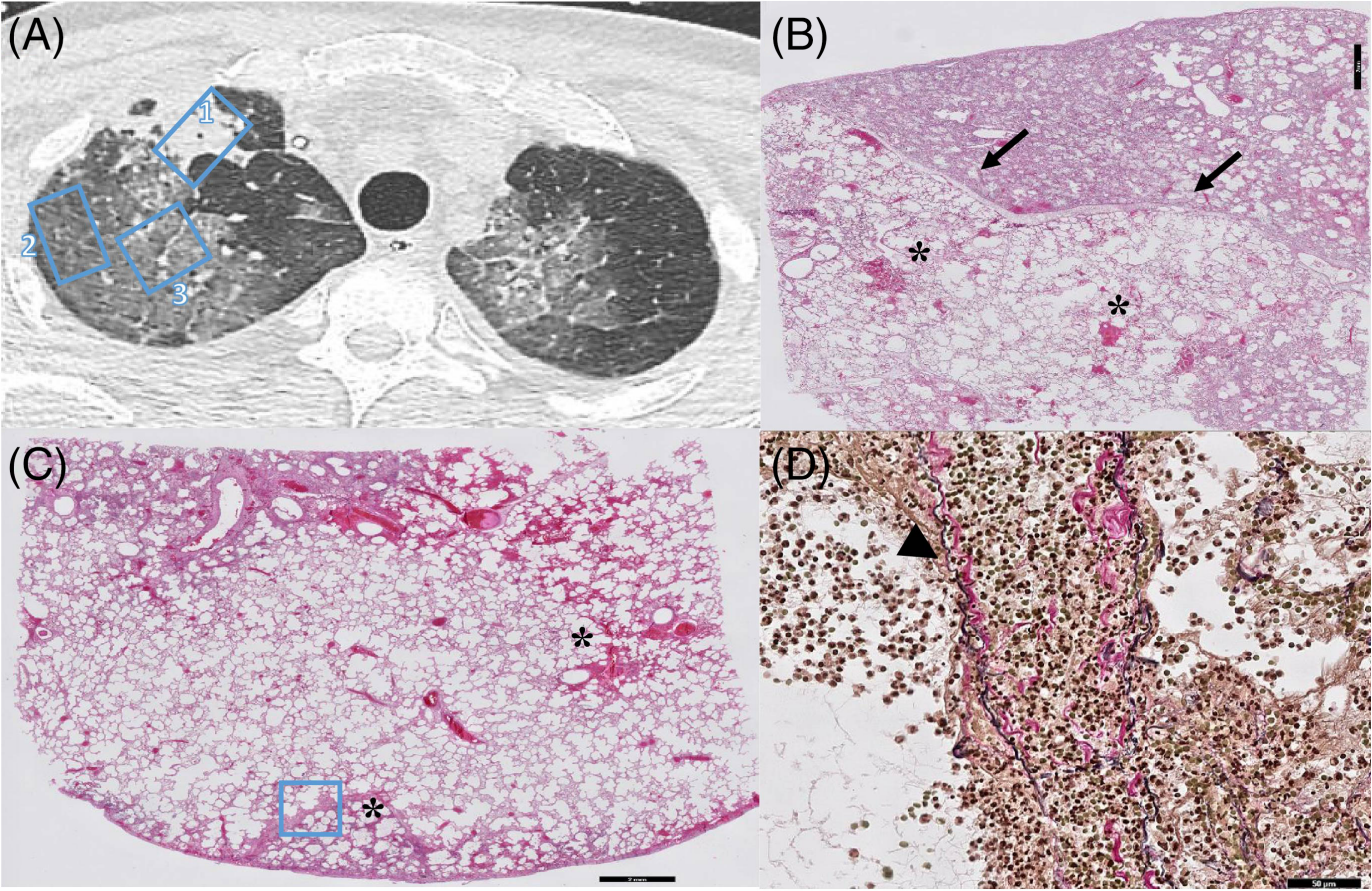
(A) CT image of the right lung reveals sharply demarcated consolidation (region 1) and diffuse GGO (region 2 and 3) with thickened interlobular septa. (B) Corresponding histopathology of sharply (arrows) demarcated consolidation (region 1) revealed acute fibrinous and organizing pneumonia (AFOP, scale bar corresponds to 2 mm). Note the microscopical haemorrhages (asterix in the spared pulmonary parenchym, scale bar corresponds to 2 mm). (C) Diffuse GGO revealed exudative stage of DAD (region 2 and 3) with microscopic haemorrhages (asterix), segmental infarction (asterix) and (D) thickened interlobular septa which were venulitis with venous thrombosis (region 3, scale bar corresponds to 50 μm)

We observed three different GGO patterns in a total of 11 sampled areas: (1) patchy GGO (*n* = 3), (2) diffuse GGO with thickened interlobular septa (*n* = 6) and/or (3) with thickened intralobular septa (crazy paving) (*n* = 2). The tissue substrates of patchy GGO were DAD (1/3), vascular damage and thrombosis (1/3) or a combination of both (1/3). Prominent interlobular septa in combination with GGO on chest CT was explained by septal lymphatic stasis and oedema (Figure 2). Diffuse GGO with thickened interlobular septa mostly corresponded to the exudative or proliferative (organizing pneumonia/organization of exudate) phase of DAD (4/6), but also harboured vascular damage and thrombosis (1/6) and a combination of both (1/6). Interestingly, one of the sampled diffuse GGO areas with radiologically thickened interlobar septa correlated with segmental infarction with venulitis and venous thrombosis (Figure 3). Both sampled areas of crazy paving (2/2) in one pancytopenic patient corresponded to exudative DAD with excessive epithelial leakage, indicating severe disruption of the endothelial barrier without any immune response. Interestingly, the consolidative areas on chest CT in this patient were histologically based on prominent oedema without inflammation or organization.

### Tissue substrates of consolidation: From oedema to fibrosis

**FIGURE 4:**
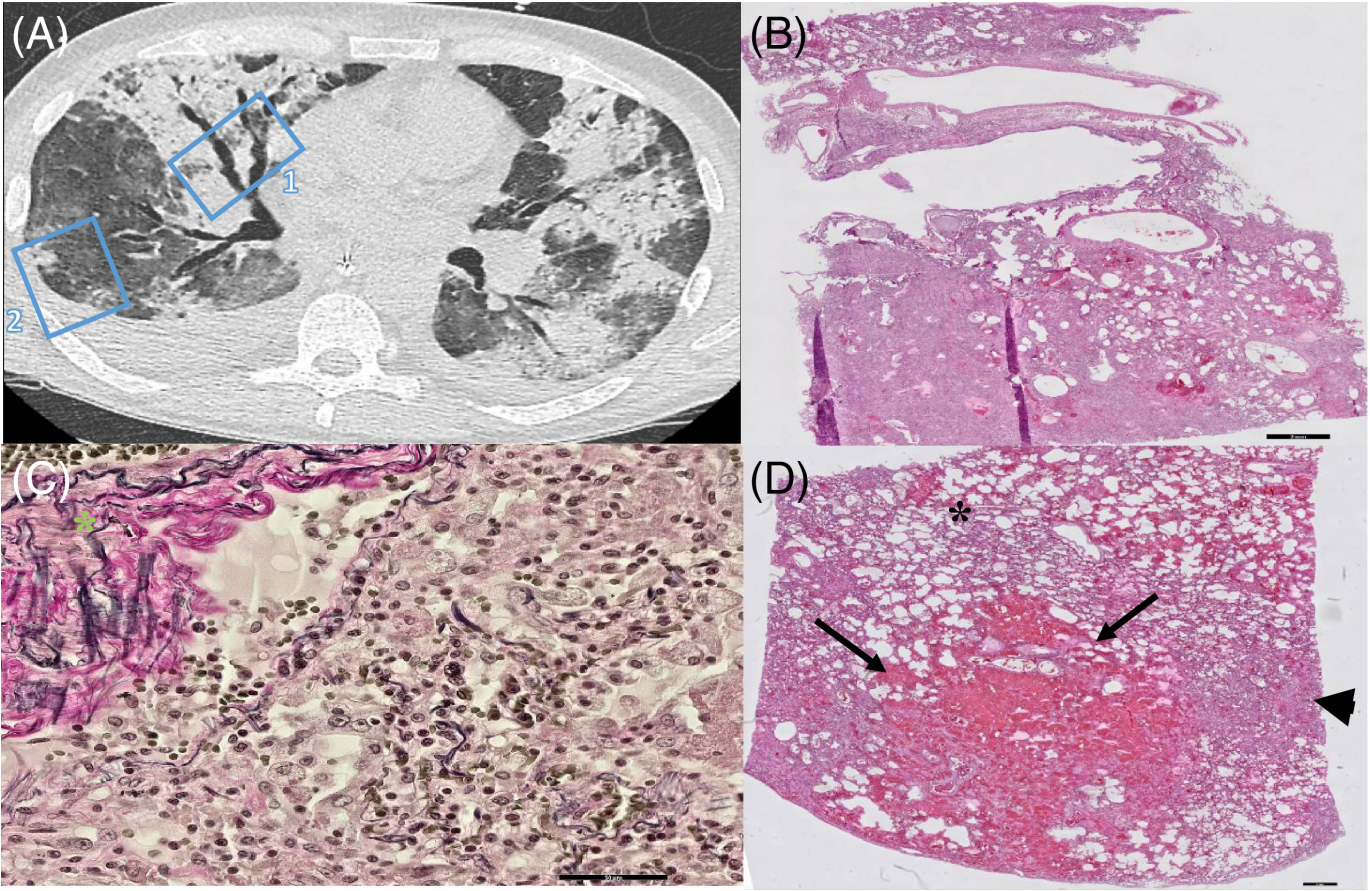
(A) CT image of the right lung reveals consolidation with traction bronchiectasis in the middle lobe (region 1) and consolidation with surrounding GGO in the right lower lobe (region 2). (B) Corresponding Histopathology of the consolidation with traction bronchiectasis in the middle lobe (region 1, scale bar corresponds to 2 mm) revealed proliferative stage of DAD. (C) EVG staining of proliferative DAD in region 1 lacking pink collagen deposition in the parenchyma indicating no fibrosis and possible reversibility of parenchymal remodelling. Note the pink collagen of normal vascular adventitia (green asterix, scale bar corresponds to 50 μm). (D) Consolidation with surrounding GGO (region 2) revealed large area of haemorrhage (arrows) with surrounding patchy microscopical haemorrhages (asterix) and exudative phase of DAD (arrowhead, scale bar corresponds to 1 mm)

We observed three different patterns of consolidation in 16 sampled areas: (1) (band‐like) consolidation with or without surrounding GGO (*n* = 11), (2) sharply demarcated consolidation (*n* = 1) and (3) consolidation with traction bronchiectasis (*n* = 4). Sampled areas of (band‐like) consolidation revealed organizing DAD (3/11), a combination of DAD, vascular damage and thrombosis (4/11) or bronchopneumonia (4/11). Especially in cases with a short disease duration (5–6 days), peribronchovascular consolidation was histologically mirrored by a prominent neutrophil infiltration (bronchopneumonia) indicating an early innate inflammatory response. Severe acute respiratory syndrome coronavirus 2 (SARS‐CoV‐2) immunohistochemistry positive cells were encountered in these regions, indicating COVID‐19 infection. Furthermore, in three out of four areas of consolidation with traction bronchiectasis, we noticed simultaneous DAD, vascular damage and thrombosis. Traction bronchiectasis is considered as a typical radiological sign of pulmonary fibrosis. However, in two of the three sampled regions with this CT pattern, histopathological findings were not accompanied by collagen deposition as demonstrated with Elastica‐van‐Gieson staining (Figure 4) indicating no fibrosis. Finally, in one area of sharply demarcated consolidation (Figure 3), we observed acute fibrinous and organizing pneumonia. This is a recently recognized distinct histological pattern of acute lung injury with a clinical presentation similar to that of classic DAD.

### Vascular damage and thrombosis in the radiologically unaffected lung

**FIGURE 5:**
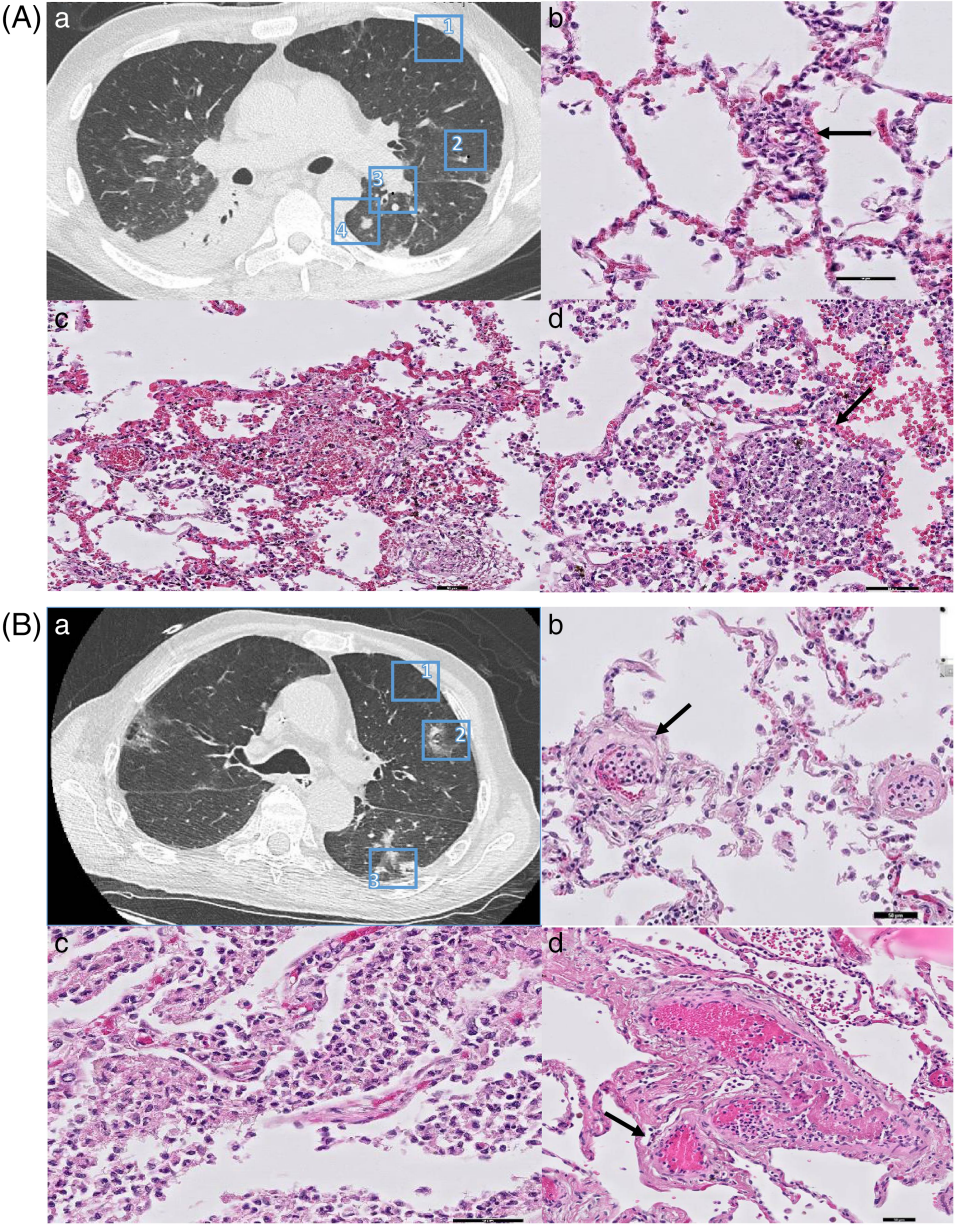
(A) CT image of the left lung reveals radiologic unaffected pulmonary parenchyma (region 1), patchy GGO (region 2), peribronchovascular consolidation (region 3) and subpleural consolidation (region 4). b. Radiologic unaffected pulmonary parenchyma (region 1) revealed patchy endothelialitis (arrow). c. Corresponding histopathology of patchy GGO (region 2) revealed patchy haemorrhages d. Corresponding histopathology of the peribronchovascular and subpleural area (region 2 and 3) revealed acute exudative pneumonia (bronchopneumonia) with neutrophil granulocytes infiltration of alveolar spaces (arrow, scale bars corresponds to 50 μm). (B) a. CT image of the left lung reveals an early stage patient with areas of unaffected pulmonary parenchyma (region 1), peribronchovascular consolidation (region 2) and subpleural consolidation (region 3). b. Corresponding histopathology of the unaffected pulmonary parenchyma (region 1) reveals early stage microthrombi (arrow). c. Corresponding histopathology of peribronchovascular consolidation (region 2 and 3) revealed bronchopneumonia with bronchocentric distribution and vascular involvement d. with thrombi (scale bars correspond to 50 μm)

Surprisingly, we observed endothelialitis (endothelial cell injury, Figure 5A, panel B) or microthrombi (Figure 5B, panel B) in two samples from the radiologically normal lung. The patients with the diagnosis of acute pulmonary embolism did not correspond to those with endothelialitis in the radiologically normal lung. A similar pattern of endothelialitis, microthrombi and microhaemorrhage was observed in the spared pulmonary parenchyma in the context of patchy GGO on chest CT, even in the absence of other histopathological signs of tissue damage.

## DISCUSSION

In this study, we present the first direct regional comparison of the most frequently observed pulmonary CT patterns with corresponding histopathological patterns in an autopsy cohort of eight COVID‐19 patients. Disease duration and causes of death varied between patients. This study provides a comprehensive and detailed correlation between radiology and pathology which is of value to the clinical interpretation of chest CT in COVID‐19 patients. We demonstrated that the typical CT findings encountered in COVID‐19 are not strictly correlated with specific histopathological patterns. Moreover, we showed that regardless of the radiological pattern, DAD and vascular damage and thrombosis are ubiquitously present in the COVID‐19‐infected lung.

DAD has been proposed as the main histopathological response in COVID‐19.^15^, ^16^, ^17^ Recently, DAD was also shown to be present in COVID‐19 patients who die in the community, suggesting no contribution of hyperoxaemic mechanical ventilation to the histological changes.^18^ Radio‐histopathological correlation studies performed before the era of COVID‐19 have already suggested that acute lung injury/DAD is responsible for most typical chest CT findings in ARDS.^19^, ^20^, ^21^ In line with these studies, we confirm that different stages of DAD in addition to vascular damage and thrombosis are key tissue substrates of the different CT patterns in COVID‐19.^6^, ^7^, ^8^, ^9^, ^10^, ^11^, ^22^ Moreover, both DAD and vascular damage and thrombosis were ubiquitously present in the COVID‐19‐infected lung, indicating that vascular damage and thrombosis contribute to the typical radiological findings in COVID‐19 as well.

However, after direct one‐to‐one correlation, we did not observe unique histopathological patterns explaining the different radiological findings. For example, consolidation corresponded to organization of exudative DAD, haemorrhage or prominent neutrophil infiltration (bronchopneumonia). This is in line with studies in acute interstitial pneumonia demonstrating that HRCT findings were not specific for the pathological findings.^23^ However, other radio‐histopathological correlation studies in interstitial lung disease demonstrated that areas of GGO with no concomitant radiological signs corresponded well to inflammation.^24^ Furthermore, it has been shown that CT characteristics allowed to distinguish between the various histological subtypes of non‐specific interstitial pneumonia.^25^

Remarkably, extensive pulmonary vascular damage was present in the radiologically unaffected pulmonary areas and in the histological spared pulmonary parenchyma. This agrees with earlier findings that vascular damage can occur during the early phase of symptomatic COVID‐19 infections.^22^ Possibly, these vascular changes precede the development of DAD. Vascular changes may also explain the frequent clinical observation of a disturbed gas exchange in a patient with a relatively normal chest CT. Vascular damage (including endothelial injury and microthrombi) has been reported in all stages of ARDS^26^ and histological tissue damage patterns in COVID‐19 patients are partially similar to lung injury of any cause as we showed in the present study. However, the extent of microthrombi in COVID‐19 ARDS was shown to be nine times as prevalent compared to patients with influenza ARDS.^10^ In addition, the amount of angiogenesis was 2.7 times as high as in patients with influenza,^10^ suggesting vascular changes are specific in COVID‐19 and an important determinant of the CT findings, as demonstrated in this study.

Taken together, we demonstrate that GGO and consolidation cannot only be attributed to DAD, but there is a significant role for vascular damage and thrombosis as well consisting of endothelial cell injury, haemorrhage and thrombosis.

Beyond the common limitations of an observational study, the presented data are based on the assumption that little or nothing changed in the period between the last scan and the autopsy. Median time between the CT scan and death was 1 day. This is the best correlation that can be achieved in an autopsy cohort. The cohort described here had a heterogeneity in patient population and duration of COVID‐19 and, because of the nature of this study, was restricted to a subpopulation of severe COVID‐19 (six of eight patients). As our cohort was largely skewed to a subpopulation of severe COVID‐19 patients, it is plausible that the histological findings of early or milder disease are missed. However, in contrast to most autopsy studies, our population included patients diverse in duration of COVID‐19 symptoms (early disease) and cause of death; we think that some of the histological findings are representable for early and milder disease as well.

In addition, due to the small sample size, no association could be determined between histopathology, clinical parameters, efficacy of therapeutics or invasive ventilation.

In conclusion, we show that the radiological patterns in COVID‐19 correspond to DAD, vascular damage and thrombosis or a combination of both. Nonetheless, the different typical CT patterns in COVID‐19 are not related to specific histopathological patterns.

## AUTHOR CONTRIBUTIONS

**Azar Kianzad:** Conceptualization; data curation; formal analysis; methodology; visualization; writing‐original draft; writing‐review & editing. **Lillian Meijboom:** Conceptualization; data curation; formal analysis; investigation; methodology; supervision; visualization; writing‐original draft; writing‐review & editing. **Esther Nossent:** Conceptualization; data curation; formal analysis; investigation; methodology; supervision; writing‐original draft; writing‐review & editing. **Eva Roos:** Data curation; formal analysis; investigation; writing‐original draft. **Bernadette Schurink:** Data curation; formal analysis; investigation; writing‐original draft. **Peter Bonta:** Data curation; formal analysis; investigation; supervision; writing‐original draft; writing‐review & editing. **Inge van den Berk:** Data curation; formal analysis; investigation; supervision; writing‐original draft; writing‐review & editing. **Rieneke Britstra:** Data curation; formal analysis; investigation; supervision; writing‐original draft; writing‐review & editing. **Jaap Stoker:** Conceptualization; data curation; formal analysis; methodology; supervision; writing‐original draft; writing‐review & editing. **Anton Vonk Noordegraaf:** Conceptualization; formal analysis; methodology; supervision; writing‐original draft; writing‐review & editing. **Paul van der Valk:** Conceptualization; data curation; formal analysis; investigation; methodology; supervision; writing‐original draft; writing‐review & editing. **Erik Thunnissen:** Conceptualization; data curation; formal analysis; methodology; supervision; writing‐original draft; writing‐review & editing. **Marianna Bugiani:** Conceptualization; data curation; funding acquisition; investigation; methodology; supervision; writing‐original draft; writing‐review & editing. **Harm Jan Bogaard:** Conceptualization; formal analysis; investigation; methodology; supervision; writing‐original draft; writing‐review & editing. **Teodora Radonic:** Conceptualization; formal analysis; investigation; methodology; supervision; writing‐original draft; writing‐review & editing.

## CONFLICT OF INTEREST

Prof. Dr Anton Vonk Noordegraaf is supported by the Netherlands CardioVascular Research Initiative (CVON‐2012‐08 PHAEDRA, CVON‐2017‐10 DOLPHIN‐GENESIS) and the Netherlands Organization for Scientific Research (NWO‐VICI: 918.16.610). He also received speaker's money from Johnson & Johnson, and Ferrer in the past 3 years, and served as a member of the scientific advisory board of Acceleron. The other authors report no conflict of interests.

## HUMAN ETHICS APPROVAL DECLARATION

The Medical Ethics Review Committee of the VU University Medical Centre (2020.167) confirmed that this study did not fall within the scope of the Medical Research Involving Human Subjects Act, as the diagnostic procedures were performed for clinical purposes. Patient consent was waived by the Medical Ethics Review Committee of the VU University Medical Centre for the same reason.

## Supporting information

**Appendix S1.** Supporting Information (Part 1).

**Appendix S2.** Supporting Information (Part 2).

**Appendix S3.** Supporting Information (Part 3).
