# Supplementary material for: COVID‐19: Histopathological correlates of imaging patterns on chest computed tomography
Source: Respirology. 2021 Jun 22;26(9):869–77. doi: 10.1111/resp.14101 (PMC8447040; doi:10.1111/resp.14101)
Supplement: Supplementary file 1 — Appendix S1. Supporting Information (Part 1). [file RESP-26-869-s003.doc]

**SUPPORTING INFORMATION (Part 1)**

**COVID-19: Histopathological correlates of imaging patterns on chest CT**

Azar Kianzad MD ^1^ , Lilian J. Meijboom MD ^2^, Esther J. Nossent MD ^1^, Eva Roos MD ^3^, Bernadette Schurink MD ^3^, Peter I. Bonta MD ^4^, Inge A.H. van den Berk MD ^5^, Rieneke Britstra MD ^3^, Jaap Stoker MD ^2^, Anton Vonk Noordegraaf MD ^1^, Paul Van der Valk MD ^3^, Erik Thunnissen ^3^, Marianna Bugiani MD ^3^, Harm-Jan Bogaard MD ^1^ & Teodora Radonic MD ^3^

1 Department of Pulmonary Medicine, Amsterdam Cardiovascular Scienes, Amsterdam UCM, Vrije Universiteit Amsterdam. De Boelelaan 1117; 1081 HV Amsterdam, the Netherlands

2 Department of Radiology and Nuclear Medicine, Amsterdam Cardiovascular Sciences, Amsterdam UCM, Vrije Universiteit Amsterdam. De Boelelaan 1117; 1081 HV Amsterdam, the Netherlands

3 Department of Pathology, Cancer Centre Amsterdam, Amsterdam UMC, Vrije Universiteit Amsterdam.. De Boelelaan 1117; 1081 HV Amsterdam, the Netherlands

4 Department of Pulmonary Medicine, Amsterdam UMC, AMC, Meibergdreef 9 1105 AZ Amsterdam, the Netherlands

5 Department of Radiology and Nuclear Medicine, Cancer Centre Amsterdam, Amsterdam UMC, AMC, Meibergdreef 9, 1105 AZ Amsterdam,the Netherlands

**Appendix S1-** *Detailed description of histological patterns of Figure S1-8*

**Figure 1: Patient 1**
**Fig. 1a** **A.** CT image of the left lung reveals *peribronchovascular consolidation and consolidation with surrounding GGO*. No gross picture was available. **B.** Histopathological examination of the LUL revealed bronchopneumonia with bronchocentric distribution and a spread in surrounding alveoli. **C.** In this early phase of inflammation vascular involvement with microthrombi was noted in the surrounding lung parenchyma.
**Fig. 1b**
**A.** *Subpleural consolidation* in the left lower lobe (LLL). **B.** Histopathological examination of the LLL also revealed bronchopneumonia. In the LLL bronchopneumonia was more prominently present compared to the LUL. Note the initial host response to the infection with numerous neutrophil granulocytes (panel C and D). E. Sars-Cov-2 immunohistochemistry positive cells were seen in bronchocentric distribution, indicating COVID-19 infection.

**Fig 1c** **A**. *Radiological unaffected pulmonary parenchyma*. **B.** Histopathological examination reveals early stage thrombus with neutrophil granulocytes.

**Figure 2: Patient 2**

**Fig. 2a** **A***.* CT image of the right lung *reveals areas of Patchy GGO* (region 1 and 2) *and Consolidation with surrounding GGO* (region 3 and 4). **B.** Correlating grossing patterns.
**Fig. 2b** **A.** *Patchy GGO in the right upper lobe (RUL*) **B.** Histopathological examination of the RUL (region 1,2) revealed a variable amount of interstitial lymphoplasmocytic inflammation with subtle (still interstitially located) organization of the exudate (proliferative stage DAD)*.* Note also the partially preserved air content. Panel **C en D** corresponds to the red and blue frame in panel B. **C.** prominent giant cell component of the inflammatory infiltrate which was of histiocytic origin (brown staining in CD68), in contrast to the until now reported giant cell pneumocytes type 2. TTF1 was negative in these cells (data not shown)*.* **D.** *Between the areas with patchy GGO, an area of spared lung parenchyma* in the RUL was observed. Histological examination demonstrates thrombi in the microcirculation (with prominent lymphocytic infiltrate of the vascular wall indicating an important endothelialitis component. **E.** Histological examination of *Patchy GGO* with prominent interlobular septa on the chest. Loose interstitial inflammation and oedema with prominent thrombotic microangiopathy is observed. **F.** Note the prominent secondary lobules septa due to lymphatic stasis and oedema.

**Fig 2c**
**A.** *Consolidation with surrounding GGO* in the dorsal areas (region 3,4). **B. C** Histological examination, shown in panel B and C, revealed dense oedema with prominent microscopical haemorrhages and a still subtle organization of the exudate (Exudative stage DAD). Note the grossing pattern of these two consolidative areas which had the same colour of the parenchyma as the normal lung but were consolidative upon palpation (fig 2 a, panel B)

**Figure 3A-B: Patient 3**

**Fig. 3a** **A.** CT image of the right lung shows *Sharply demarcated consolidation (1) and diffuse GGO (region 2,3) with thickened interlobular septa*. **B.** Correlating grossing patterns.  **Fig 3b**
**A.B** Histopathological examination of the *sharply demarcated consolidation* (region 1) revealed acute fibrinous and organizing pneumonia (AFOP). Note the microscopical haemorrhages (asterix) and thrombus in an area of spared lung parenchyma in the radiological unaffected parenchyma as demonstrated in panel A and in detail in panel C.
**Fig 3c**
**A.** Histopathological examination of *Diffuse GGO* with thickened interlobular septa (region 2) revealed exudative stage of diffuse alveolar damage (DAD) with microscopic haemorrhages (asterix) and segmental infarction (asterix). Blue framed area of panel A is shown in detail in panel **C:** Background parenchyma was necrotic with loss of nuclear detail of pneumocytes and capillaries and hyaline membranes covering alveolar septa.
**B.** Histopathological examination of *Diffuse GGO* with thickened interlobular septa (region 3) revealed comparable picture of exudative stage DAD with necrotic parenchyma and prominent venulitis and venous thrombosis**. D. E** Note also (detailed red frame of panel B and detailed yellow fram of panel A) the thickened interlobular septa which were histologically venulitis with venous thrombosis.

**Fig 3d
A.** CT image of the right lung revealed *Consolidation with traction bronchiectasis* in the middle lobe (ML, region 1) and *consolidation with surrounding GGO* in the right lower lobe (RLL, region 2). **B**. Correlating grossing patterns. Mirrored picture of the same level as chest CT Gross examination showed grey hepatisation of the RML and spongy accentuation of the parenchyma with haemorrhage in the RLL.

**Fig 3e
A.** Histopathological examination of the *Consolidation with traction bronchiectasis* in the middle lobe (region 1), revealed hepatisation which was a proliferative stage of DAD with prominent fibrin exudation (AFOP) but no collagen deposition or haemorrhage. Panel **B** shows the EVG staining lacking pink collagen deposition in the parenchyma in contrast to the adventitia of a vein (yellow arrow, as internal control).

**C.** histopathological examination of the *consolidation with surrounding GGO* (region 2) revealed large area of haemorrhage with surrounding patchy microscopical haemorrhages, exudate and subtle organization of exudate.

**Figure 4: Patient 4**

**Fig 4a
A.** CT image of the right lung reveals*(Subpleural) consolidations (region 1 and 3) with diffuse GGO and diffuse GGO (region 2) and in the background pre-existent emphysema*. B. Correlating grossing patterns. This was different in the three regions. Region 3 was partially haemorrhagic, region 2 had relatively soft consistency compared to 1 or 3.
**Fig 4b**
**A.** Histopathological examination of the *consolidation* in region 1 revealed prominent exudate (exudative stage DAD) and less prominent haemorrhage. **B.** Histopathological examination of the *subpleural consolidation in region 2* (in detail in panel C) revealed protein rich exudate and patchy but prominent haemorrhages. Note the interstitial inflammation and haemorrhage with partial septal lysis.

**Fig 4c**
**A.** Histopathological examination of the *Diffuse GGO* in region 3 (in detail in panel B) revealed an area of late exudative phase (proliferative stage DAD) and early organization. **C.** an area with thrombotic angiopathy in an area of organization (panel A, red frame) is observed.

**Figure 5: Patient 5**

**Fig. 5a** ***A.*** CT image of the right lung reveals *GGO with thickened intralobular and interlobular septa (crazy paving, area 1&2) with adjacent consolidation (area 3)*. **B.** Correlating grossing patterns. Grossing pattern was palpable consolidation of variable consistency.

**Fig 5b**
**A.** Histopathological examination of the *GGO in region 1 and* ***C.*** *region 2*and in detail in panel **B &D**, revealed interstitial exudate (exudative stage DAD) with preservation of air content. Note the thickened septa of the secondary and primary lobules (arrows in panel A). **B.** There was no inflammatory reaction due to pancytopenia and therefore no endothelialitis nor thrombi.
**Fig 5c**
A. In the more consolidative area on the CT (region 3) a spectrum of the same histological pattern was seen only more dense and more alveolar filling (detail in panel B).

**Figure 6: Patient 6**

**Fig. 6a** **A.** CT image of the left lung reveals *Radiologic unaffected pulmonary parenchyma (region 1), patchy GGO (region 2), and peribronchovascular consolidation (region 3) and subpleural consolidation (region 4).* B. Correlating grossing patterns.

**Fig. 6b
A.** Histological examination of the *radiological unaffected pulmonary* area in region 1, revealed patchy early stage changes, patchy endothelialitis and segregation of neutrophil granulocytes with thrombocytes (detail in B). **C.** Histological substrate of *patchy GGO* (region 2) was patchy haemorrhages (shown in panel in detail **D**) and subtle exudate. Note the lysis of alveolar septa, fibrin exudation and haemorrhage.
**Fig. 6c**
**A.** Histological examination of the and *peribronchovascular* areas (region 3) and **C.** *subpleural* (region 4), revealed acute exudative pneumonia (bronchopneumonia) with neutrophil granulocytes infiltration of alveolar spaces (**detail in B**). D. Sars-Cov2 immunohistochemistry positive cells were encounteres in these regions, indicating COVID-19 infection.

**Figure 7: Patient 7**

**Fig. 7a** **A**. CT image of the right lung revealed *whole lung involvement with Consolidation* varying in density and regions of *GGO and traction bronchiectasis.* There was a large cavitation in the RLL. No gross picture was available. Panel **B** and in detail **C&D**: Histologically a late stage proliferative and fibrotic organization of exudate was found with collagen deposition (fibrotic stage DAD).
**Fig. 7b**
**A.** EVG staining of the medium magnification showing bronchi which were extensively remodelled and collapsed in the sections). **B.** EVG staining of high magnification showing collagen deposition (pink fibres) in the parenchyma, supporting the diagnosis of fibrosing organization**. C.** Histological examination of thrombus (acute PE).

**Figure 8: Patient 8**

**Fig 8a
A.** CT image of the right lung revealed*Diffuse GGO* (region 1),*Diffuse GGO with traction bronchiectasis* (region 2) and *Subpleural consolidations with traction bronchiectasis and surrounding GGO (region 3)*. **B.** Correlating grossing patterns. **C**. Histopathological examination of diffuse GGO (region 1, detail in panel **D**) revealed progression of early exudative phase of lung (exudative stage DAD) injury with partial preservation of air content. Interstitial edema and microscopic haemorrhages were found. **F.** Histopathology of the region 2 on the CT showed a variable organization of exudate with beginning fibrosis (pink fibrillar collagen deposition, high magnification detail in **E,** arrow).

**Fig 8b**
**A.** Histopathology of *subpleural consolidation (area 3*, and in detail in panel **B)** revealed prominent loss of air content was seen with early proliferative changes of fibroblasts with a prominent lymphoplasmocytic inflammation (proliferative DAD).

**Fig 8c
A.** CT image of the right lung revealed*Band-like subpleural consolidation (region 4)* in the basal region of RUL and apex RLL, transition of *diffuse* *GGO* to spared normal lung (region 5) and *consolidation with traction bronchiectasis* (region 6). Note the atelectasis of the right lower lobe with volume loss. **B.** Gross examination revealed more extensive parenchyma involvement with no normal lung parenchyma.

**Fig 8d
A.** Histopathology of *Band-like subpleural consolidation (region 4, in detail in panel B), revealed* exudate in late proliferative stage with subtle collagen deposition (fibrotic stage DAD).
**C.** *Transition of diffuse GGO to spared pulmonary parenchyma (region 5)* revealed Diffuse protein-rich exudate (detail in D) and microscopic haemorrhages (detail in **E**) with partial preservation of air content but histologically no normal lung. **F.***Consolidation with bronchiectasis (region 6),* revealed a dense inflammation and haemorrhages with organization of exudate (Proliferative stage of DAD). Note the partially organized thrombus in segmental pulmonary artery (acute PE) (asterix). **G.** Detail of late exudative stage of lung injury with no collagen deposition.

**Figure S1***- Patient 1*

*
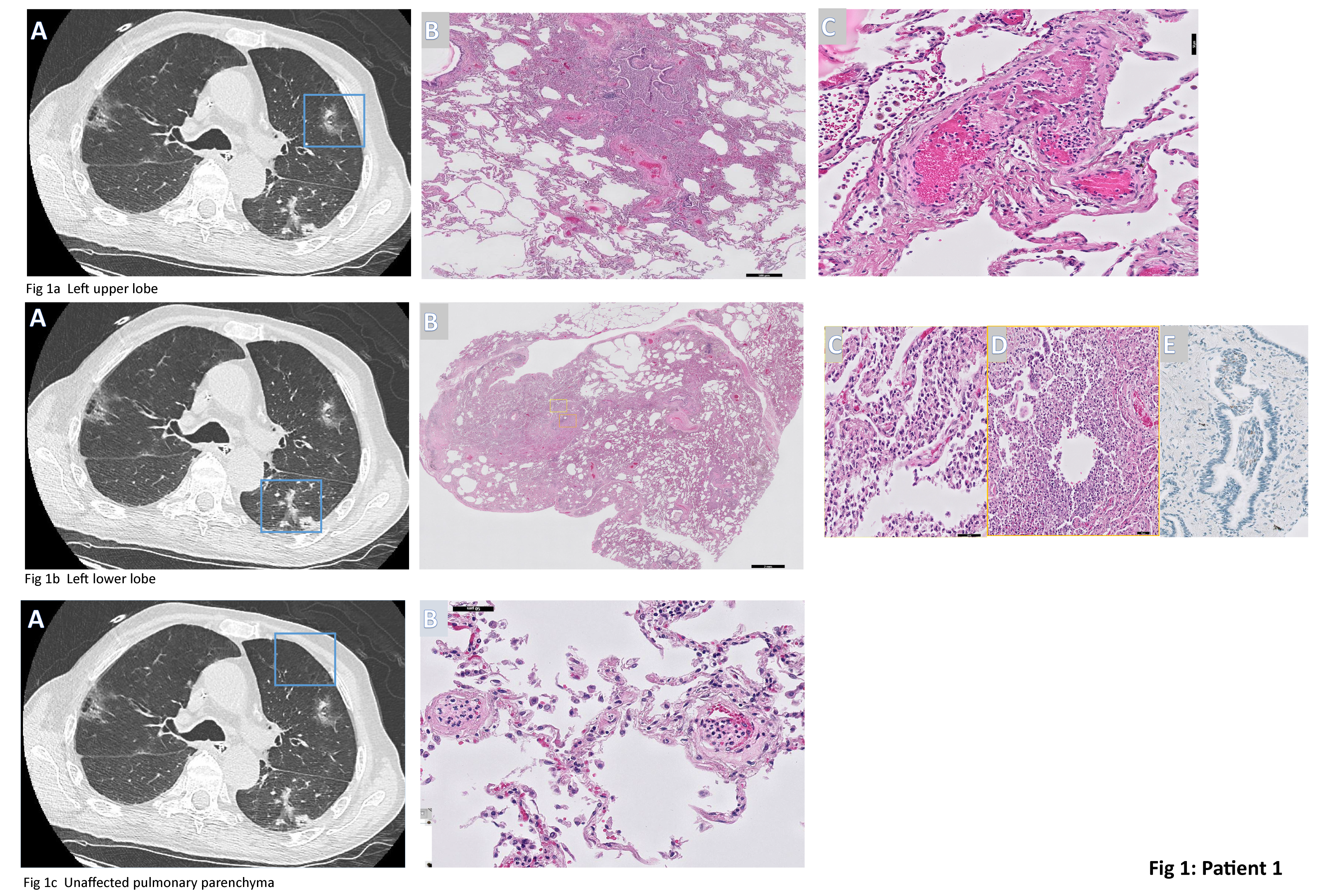
*

**Figure S2***- Patient 2*

*
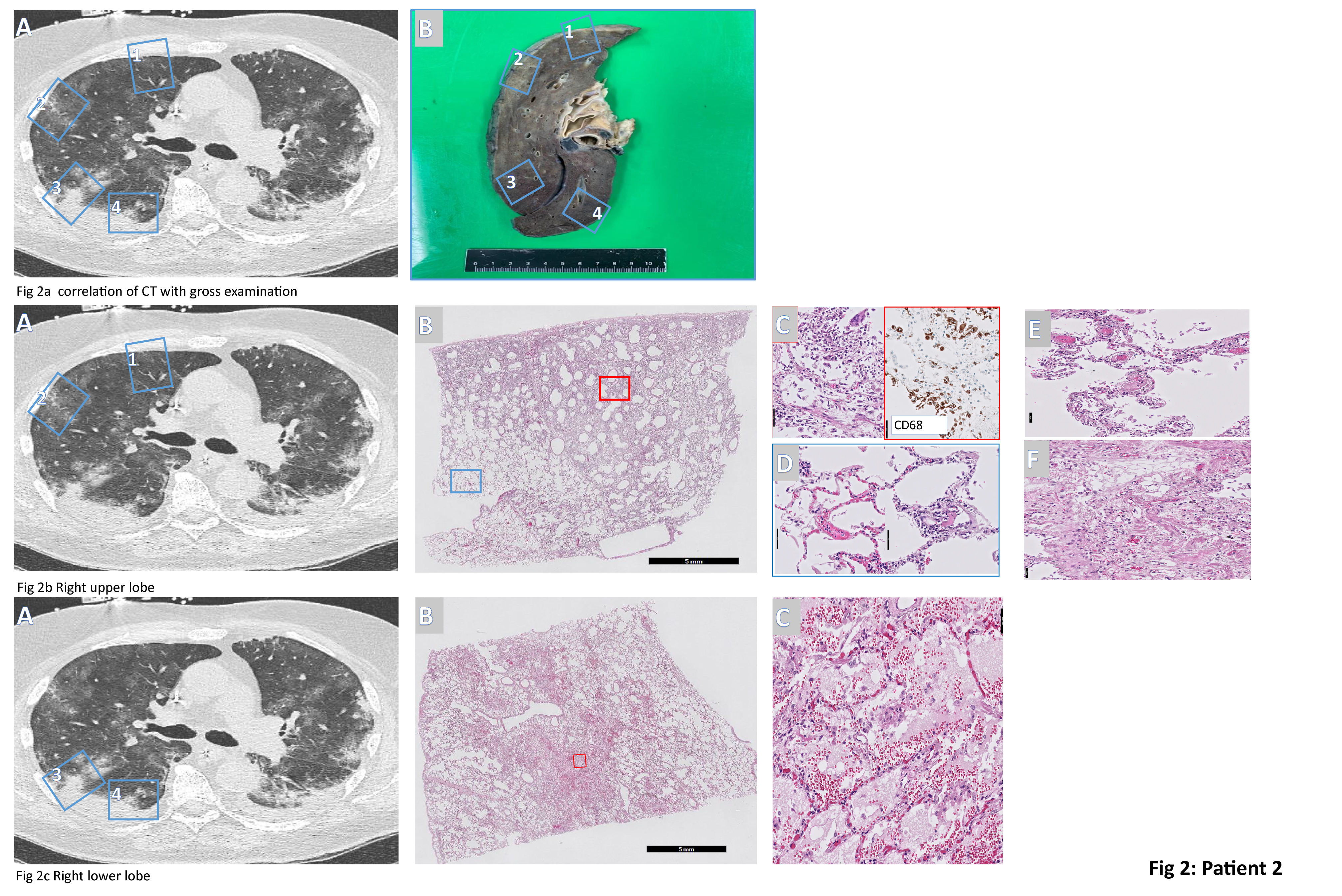
*
