# Supplementary material for: COVID‐19: Histopathological correlates of imaging patterns on chest computed tomography
Source: Respirology. 2021 Jun 22;26(9):869–77. doi: 10.1111/resp.14101 (PMC8447040; doi:10.1111/resp.14101)
Supplement: Supplementary file 2 — Appendix S2. Supporting Information (Part 2). [file RESP-26-869-s002.docx]

**SUPPORTING INFORMATION (Part 2)**

**COVID-19: Histopathological correlates of imaging patterns on chest CT**

Azar Kianzad MD ^1^ , Lilian J. Meijboom MD ^2^, Esther J. Nossent MD ^1^, Eva Roos MD ^3^, Bernadette Schurink MD ^3^, Peter I. Bonta MD ^4^, Inge A.H. van den Berk MD ^5^, Rieneke Britstra MD ^3^, Jaap Stoker MD ^2^, Anton Vonk Noordegraaf MD ^1^, Paul Van der Valk MD ^3^, Erik Thunnissen ^3^, Marianna Bugiani MD ^3^, Harm-Jan Bogaard MD ^1^ & Teodora Radonic MD ^3^

1 Department of Pulmonary Medicine, Amsterdam Cardiovascular Scienes, Amsterdam UCM, Vrije Universiteit Amsterdam. De Boelelaan 1117; 1081 HV Amsterdam, the Netherlands

2 Department of Radiology and Nuclear Medicine, Amsterdam Cardiovascular Sciences, Amsterdam UCM, Vrije Universiteit Amsterdam. De Boelelaan 1117; 1081 HV Amsterdam, the Netherlands

3 Department of Pathology, Cancer Centre Amsterdam, Amsterdam UMC, Vrije Universiteit Amsterdam.. De Boelelaan 1117; 1081 HV Amsterdam, the Netherlands

4 Department of Pulmonary Medicine, Amsterdam UMC, AMC, Meibergdreef 9 1105 AZ Amsterdam, the Netherlands

5 Department of Radiology and Nuclear Medicine, Cancer Centre Amsterdam, Amsterdam UMC, AMC, Meibergdreef 9, 1105 AZ Amsterdam,the Netherlands

**Figure S3***- Patient 3, Figure A*

*
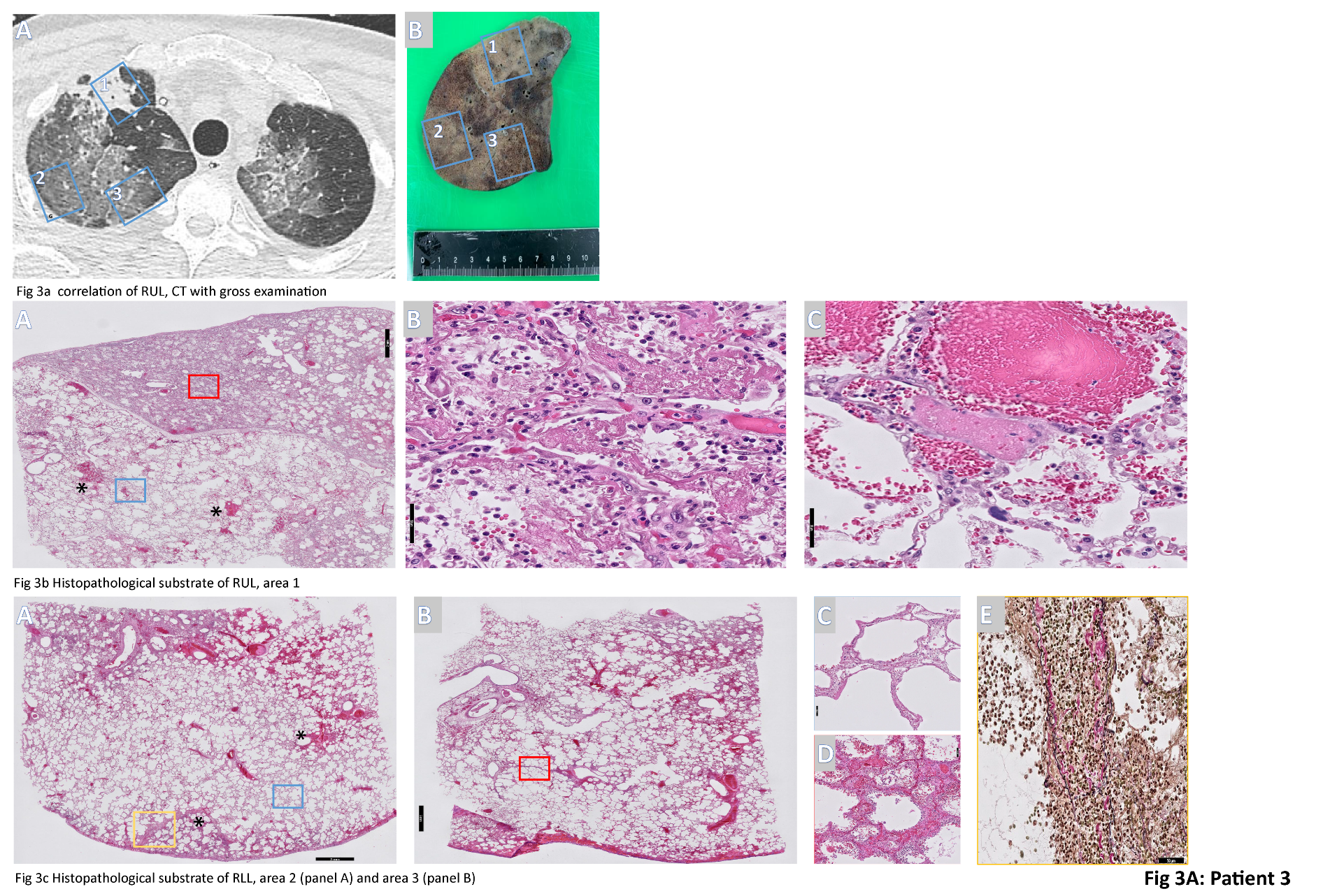
*

**Figure S3***- Patient 3, Figure B

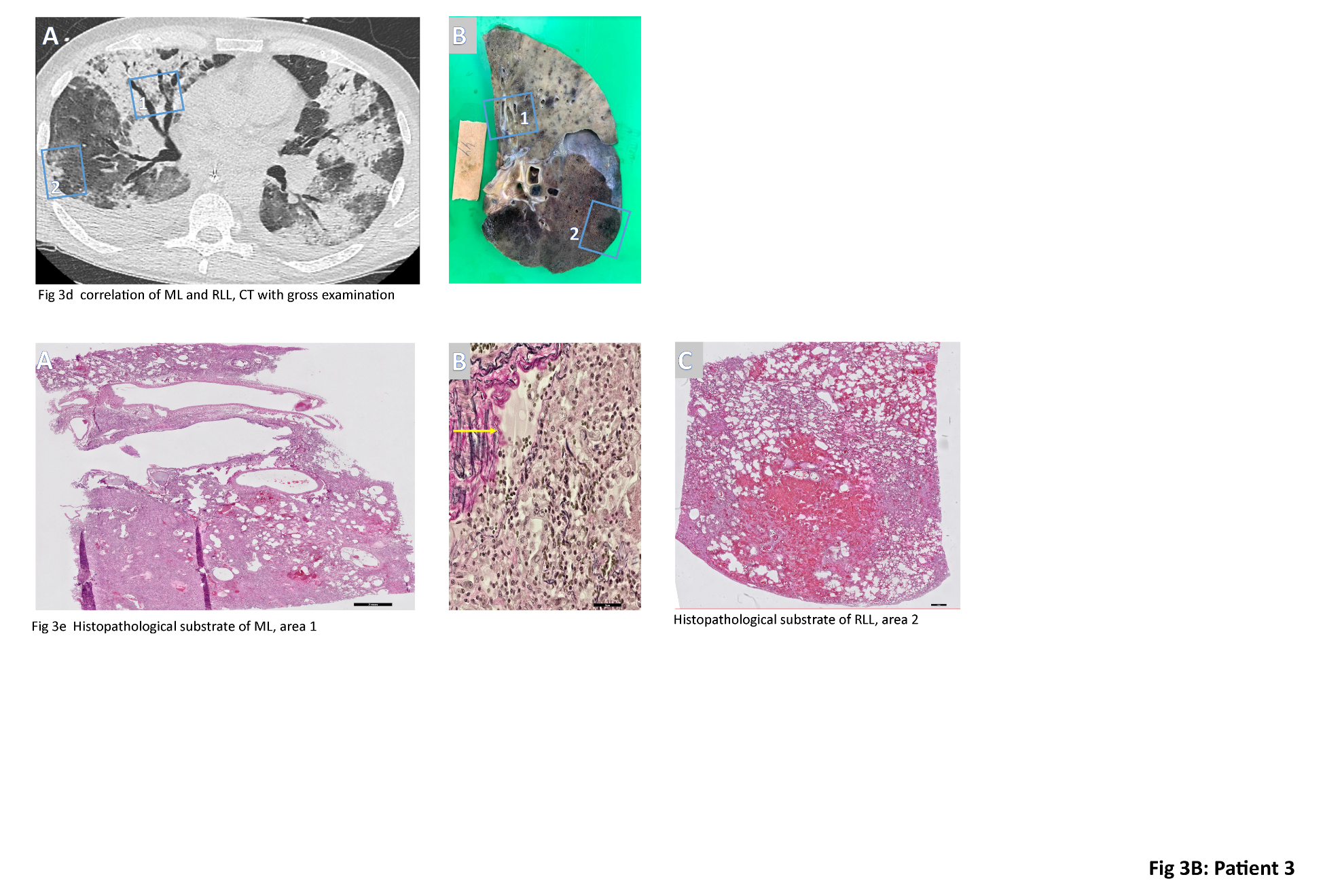
*

**Figure S4***- Patient 4

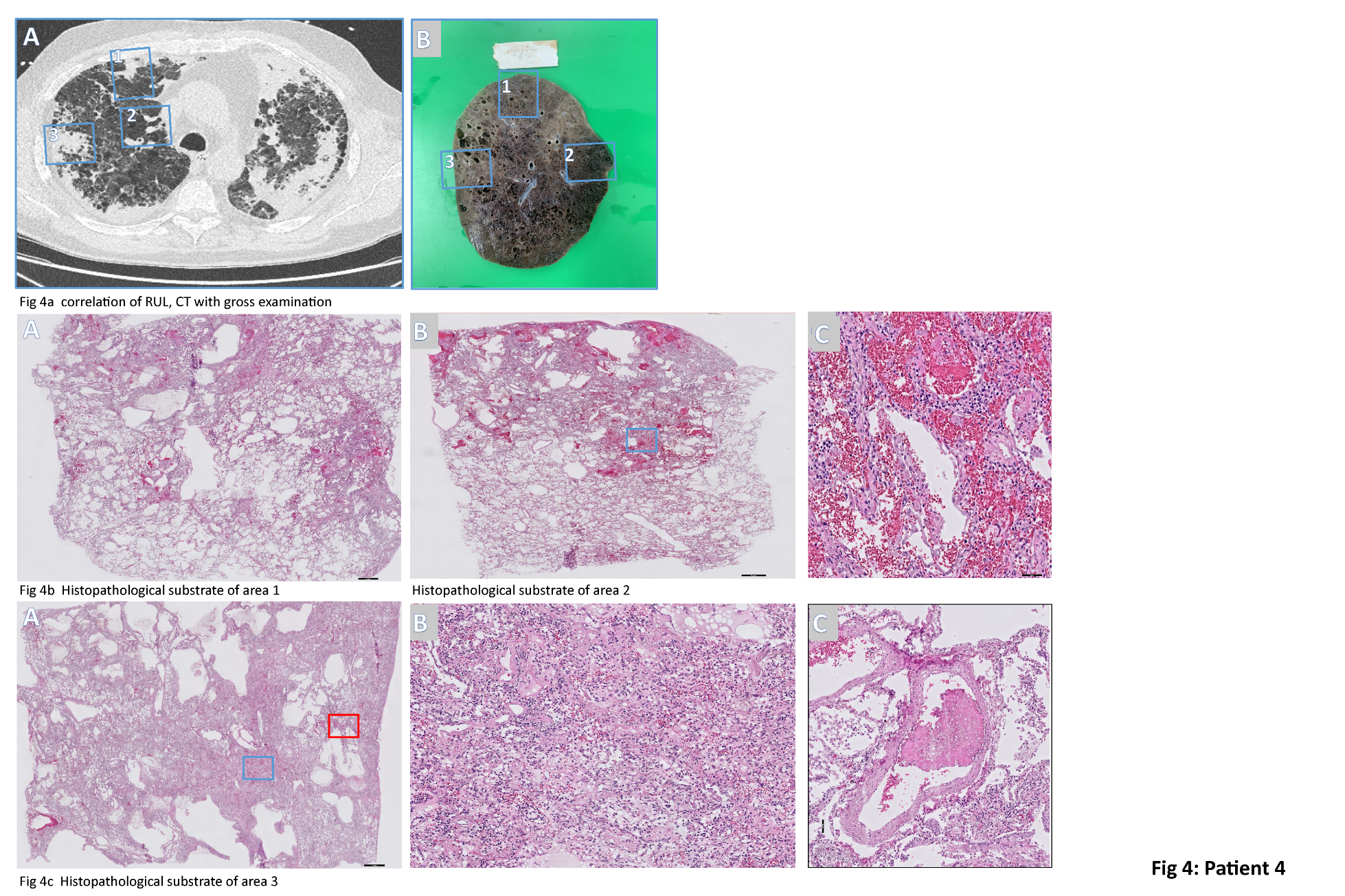
*
