# Supplementary material for: COVID‐19: Histopathological correlates of imaging patterns on chest computed tomography
Source: Respirology. 2021 Jun 22;26(9):869–77. doi: 10.1111/resp.14101 (PMC8447040; doi:10.1111/resp.14101)
Supplement: Supplementary file 3 — Appendix S3. Supporting Information (Part 3). [file RESP-26-869-s001.docx]

**SUPPORTING INFORMATION (Part 3)**

**COVID-19: Histopathological correlates of imaging patterns on chest CT**

Azar Kianzad MD ^1^ , Lilian J. Meijboom MD ^2^, Esther J. Nossent MD ^1^, Eva Roos MD ^3^, Bernadette Schurink MD ^3^, Peter I. Bonta MD ^4^, Inge A.H. van den Berk MD ^5^, Rieneke Britstra MD ^3^, Jaap Stoker MD ^2^, Anton Vonk Noordegraaf MD ^1^, Paul Van der Valk MD ^3^, Erik Thunnissen ^3^, Marianna Bugiani MD ^3^, Harm-Jan Bogaard MD ^1^ & Teodora Radonic MD ^3^

1 Department of Pulmonary Medicine, Amsterdam Cardiovascular Scienes, Amsterdam UCM, Vrije Universiteit Amsterdam. De Boelelaan 1117; 1081 HV Amsterdam, the Netherlands

2 Department of Radiology and Nuclear Medicine, Amsterdam Cardiovascular Sciences, Amsterdam UCM, Vrije Universiteit Amsterdam. De Boelelaan 1117; 1081 HV Amsterdam, the Netherlands

3 Department of Pathology, Cancer Centre Amsterdam, Amsterdam UMC, Vrije Universiteit Amsterdam.. De Boelelaan 1117; 1081 HV Amsterdam, the Netherlands

4 Department of Pulmonary Medicine, Amsterdam UMC, AMC, Meibergdreef 9 1105 AZ Amsterdam, the Netherlands

5 Department of Radiology and Nuclear Medicine, Cancer Centre Amsterdam, Amsterdam UMC, AMC, Meibergdreef 9, 1105 AZ Amsterdam,the Netherlands

**Figure S5***- Patient 5

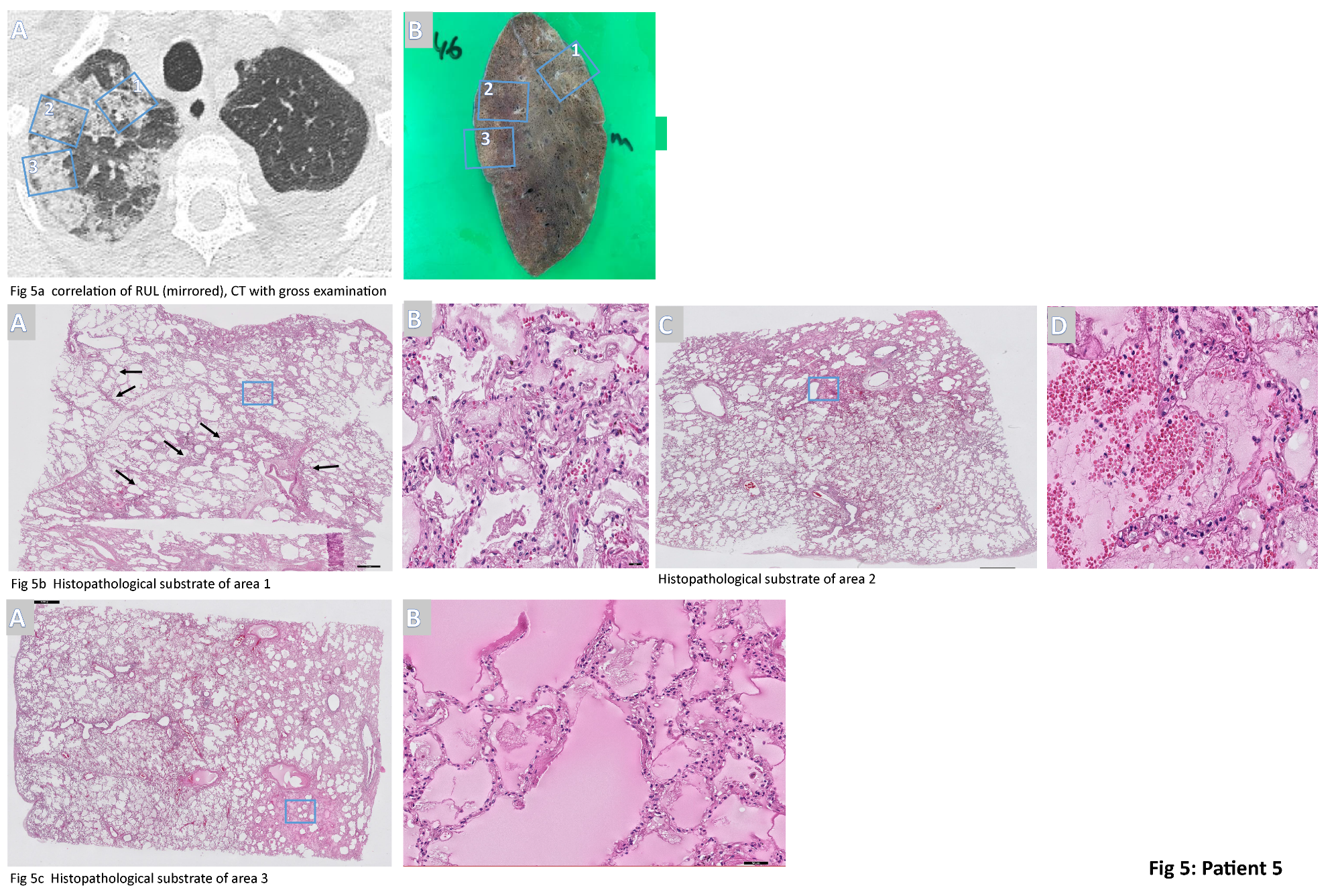
*

**Figure S6***- Patient 6

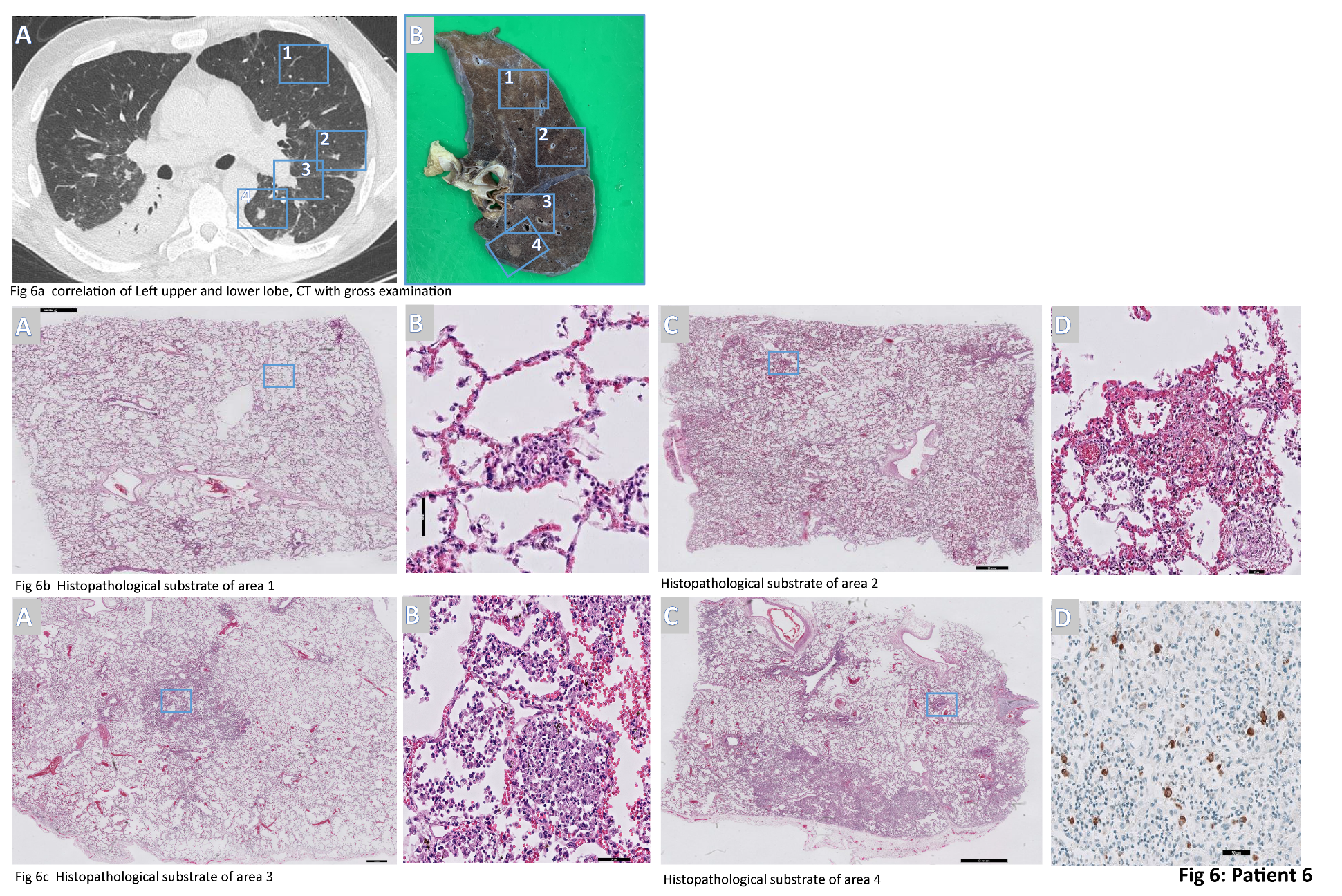
*

**Figure S7***- Patient 7*

*
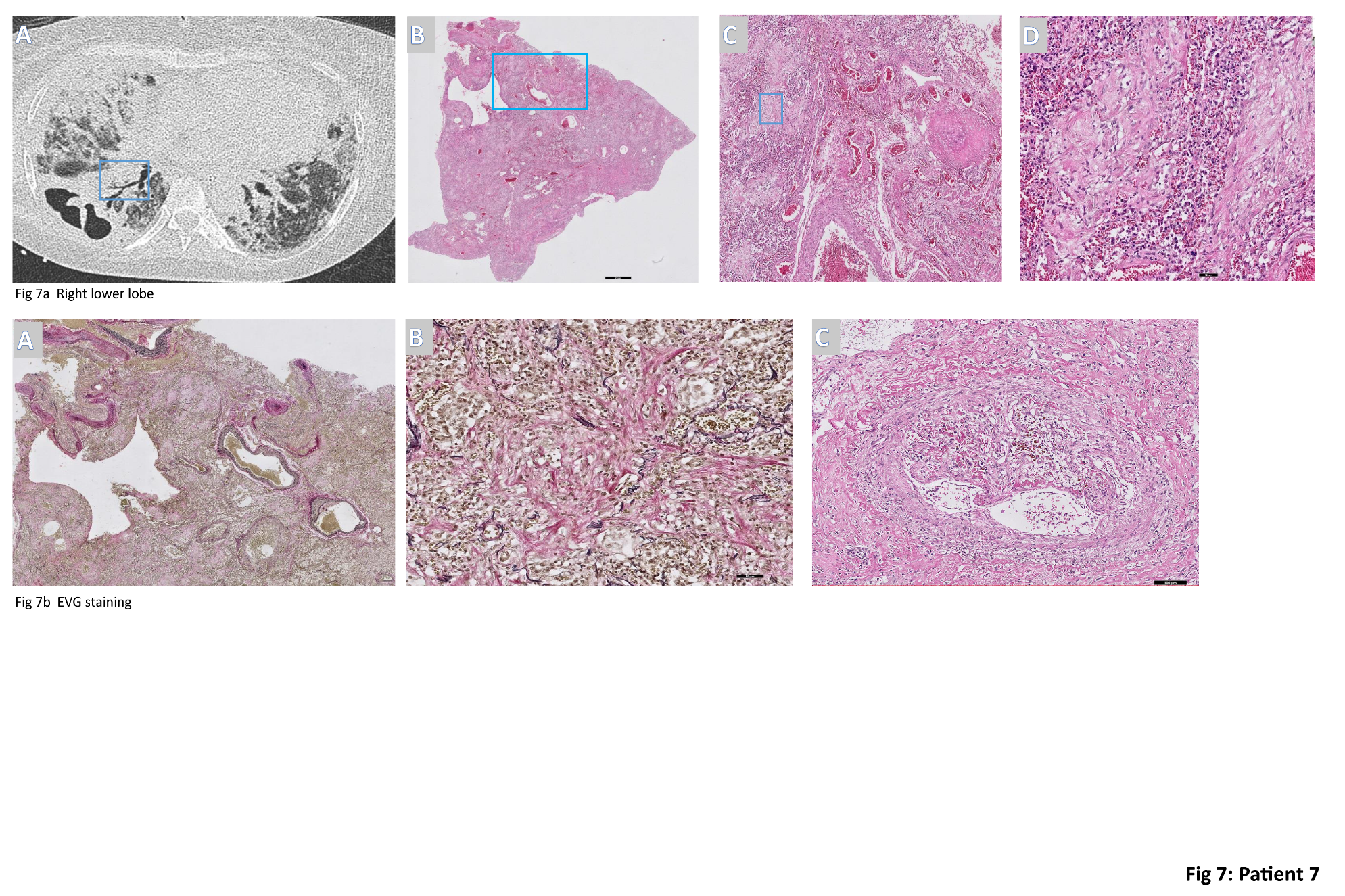
*

**Figure S8***- Patient 8

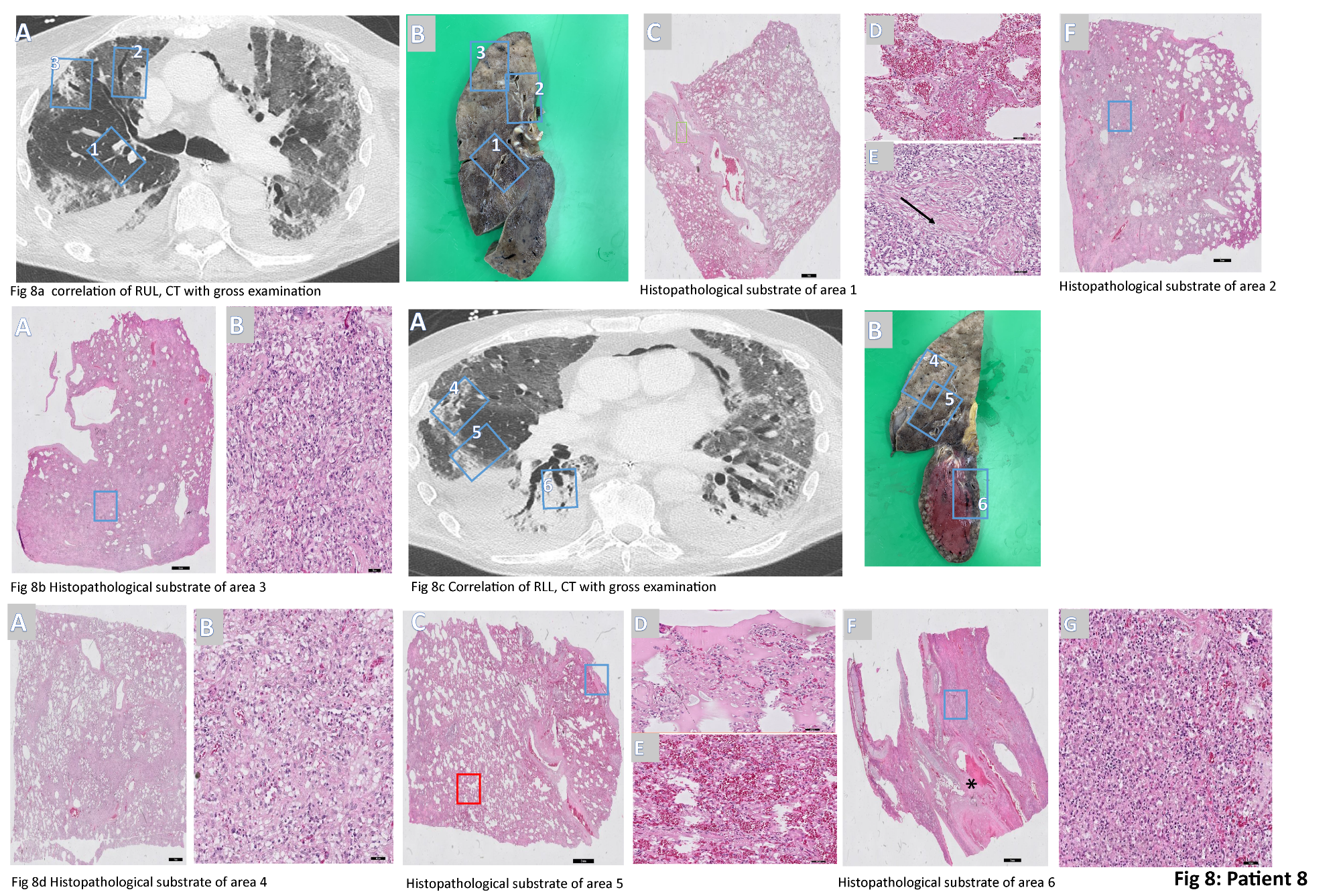
*

**Table S1-** *Baseline characteristics*

| **Patients** | 1 | 2 | 3 | 4 | 5 | 6 | 7 | 8 |
| --- | --- | --- | --- | --- | --- | --- | --- | --- |
| **Age (y)/Sex** | 73 M | 74 M | 64 M | 72 M | 67 M | 41 M | 60 F | 74 M |
| **Comorbidity** | Gastro-intestinal malignancy Hypertension | Hypertension CVA Hypercholesterolemia Angina pectoris  Type 2 Diabetes | - | Hypertension | Hematologic malignancy (pancytopenia) | Neurologic disease (not COVID-19 related) | Asthma | Hypercholesterolemia |
| **Signs and symptoms at onset of illness** | Fever, cough | Fever, cough, headache | Fever, dyspnea, cough | Fever, cough, vomiting | Fever | - | Fever, dyspnea, headache, Myalgia | Cough |
| **Duration COVID-19 symptoms until death (days)** | 5 | 22 | 14 | 17 | 10 | 6 | 30 | 44 |
| **Duration of hospitalization (days)** | 11 | 14 | 10 | 11 | 59 | 70 | 25 | 37 |
| **Invasive ventilation/ICU** | no | yes | yes | yes | no | yes | yes | yes |
| **Duration of invasive ventilation (days)** | - | 13 | 9 | 8 | - | 69 | 25 | 32 |
| **Treatment regimen** | - | Empiric antibiotics Chloroquine Lopinavir/Ritonavir | Empiric antibiotics Chloroquine | Empiric antibiotics Chloroquine | Empiric antibiotics and antifungal treatment | Prednisolon 20mg daily | Empiric antibiotics Chloroquine Lopinavir/Ritonavir | Empiric antibiotics Chloroquine  Prednisolon 80mg daily |
| **Anticoagulation treatment** | Nadroparin 2850 IE daily | Nadroparin 2850 IE daily | Nadroparin 2850 IE daily followed by Heparin infusion | Nadroparin 2850 IE daily | - | Nadroparin 2850 IE daily | Nadroparin 2850 IE daily followed by heparin infusion | Nadroparin 2850 IE daily followed by heparin infusion |
| **Cause of death** | Complication of extensive stage of malignancy | Respiratory insufficiency due to COVID-19 | Multi-organ failure due to COVID-19 | Multi-organ failure due to COVID-19 | Respiratory insufficiency due to COVID-19 | Neurological disease (non COVID-19 related) | Respiratory insufficiency due to COVID-19 | Respiratory insufficiency due to COVID-19 |
| **Dominant radiological patterns** | Peribronchovascular consolidation | Patchy GGO | Consolidation with traction bronchiectasis | Subpleural consolidation | Subpleural consolidation | Subpleural and peribroncho-vascular consolidation | Consolidation with traction bronchiectasis | Diffuse GGO |
| **% Opacity in Consolidation/GGO** | 1/5 | 12/24 | 27/39 | 24/33 | 22/40 | 7/6 | # | 24/54 |
| **Premortem CT scan used for analysis (days before death)  Patient intubated during CT scan** | 1  no | 3  yes | 2   yes | 1  yes | 2  no | 1  yes | 0  yes | 1  yes |
| **Pulmonary embolism CTPA** | unknown  (plain CT only) | unknown  (plain CT only) | Bilateral segmental and subsegmental emboli | no | unknown  (plain CT only) | no | Bilateral segmental emboli | Central and subsegmental emboli |
| **Signs of RV dysfunction - LV/RV > 1**  **- Enlarged pulmonary artery** | no no | ## no | yes (1,3) no | yes (1.26) yes (30 mm) | ## no | no yes (31 mm) | no yes (28 mm) | No  yes (30 mm) |

LV = left ventricle; RV = right ventricle;

# the automatic software didn’t yield measurable percentages in this patient. Visually both lungs were almost completely consolidated with only small areas of GGO

## no contrast was administered

**Table S2-** *Tissue substrates of dominant radiological patterns*

| **DOMINANT RADIOLOGICAL PATTERNS** | **SAMPLED AREAS (n)** | **DAD (n)** | **VASCULAR DAMAGE AND THROMBOSIS (n)** | **COMBINED (n)** | **BRONCHO-**  **PNEUMONIA (n)** |
| --- | --- | --- | --- | --- | --- |
| **Unaffected pulmonary parenchyma** | 2 | 0 | 2 | 0 | 0 |
| **Patchy GGO** | 3 | 1 | 1 | 1 | 0 |
| **Diffuse GGO with thickened interlobular septa** | 6 | 4 | 1 | 1 | 0 |
| **Crazy paving** | 2 | 2 | 0 | 0 | 0 |
| **Consolidation** | 16 | 5* | 0 | 7 | 4 |
| **Total** | 29 | 12 | 4 | 9 | 4 |

DAD = diffuse alveolar damage; GGO = ground glass opacity;
Data is presented as numbers.

*consisting of one patient with AFOP (acute fibrinous and organizing pneumonia)
